# Data on accumulative allocation of water rights in the Atacama Desert (Antofagasta Region, northern Chile), 1905–2018

**DOI:** 10.1016/j.dib.2022.108296

**Published:** 2022-05-21

**Authors:** Matías Calderón-Seguel, María Christina Fragkou, Rodrigo Fuster, Francisco Mayol, Manuel Prieto

**Affiliations:** a Departamento de Ciencias Históricas y Geográficas, Universidad de Tarapacá, 18 de septiembre 2222, Arica 1010069, Chile; b Departamento de Geografía, Universidad de Chile, Portugal 84, Santiago 8331051, Chile; c Departamento de Ciencias Ambientales y Recursos Naturales Renovables, Laboratorio de Análisis Territorial, Facultad de Ciencias Agronómicas, Universidad de Chile, Santa Rosa 11315, La Pintana, 8820808, Chile

**Keywords:** Water privatization, Water governance, Extractivism, Chilean water model, Loa River, Atacama Salt Flat

## Abstract

This article presents a dataset on the accumulated water flow (L/s) granted in the Antofagasta Region for each year between 1905 and 2018. We produced the dataset starting from the official public records on water rights (*Registro Público de Derechos de Aprovechamiento de Aguas,* RPDAA), which are free to access and available at the National Water Agency's website (*Dirección General de Aguas*, DGA). The initial data described 1047 individual water rights granted in the Antofagasta Region according to 65 criteria. In order to find errors in the data, inconsistencies between the data, or/and the absence of relevant information, we revised and validated the data through different methods, including a literature review and interviews to public officials. Then, we calculated the accumulated water flow (L/s) from the annual flow granted each year (1905–2018) in the two main basins of the region: the Loa River Basin, and the Salar de Atacama Basin. In doing so, we differentiated the type of water (ground or surface water) and the use of water. Thus, the data show and compare temporal variations in the allocation of ground and surface water to different water uses in the two basins. The data are useful to researchers, decision makers and to the general population interested in the processes of water distribution within the Chilean context.

## Specifications Table

**Table:** 

Subject	Social Sciences
Specific subject area	Geography, Political Ecology, Water Management
Type of data	Table
How the data were acquired	We downloaded the initial data from the official public records on water rights (*Registro Público de Derechos de Aprovechamiento de Aguas*, RPDAA), which are freely available at the website of the Chilean Water Agency (*Dirección General de Aguas*, DGA): https://dga.mop.gob.cl/productosyservicios/derechos_historicos/Paginas/default.aspx (accessed 11 May 2022)
Data format	Raw
Description of data collection	We downloaded the official public records on water rights for the region (Antofagasta) as an excel file (30/04/2019). This file contained a table with 1047 records of water rights granted in the Antofagasta Region between 1905 and 2018, described according to 65 criteria. This data was processed as it is described below in order to create the database presented in this article.
Data source location	*• Institution:* Chilean Water Agency *(Dirección General de Aguas*, DGA) *• Country:* Chile
Data accessibility	Repository name: Mendeley data Data identification number: DOI:10.17632/vx93gm8zgb.2 Direct URL to data: https://data.mendeley.com/datasets/vx93gm8zgb/2
Related research article	M. Prieto, M. Calderón-Seguel, M.C. Fragkou, R. Fuster, The (not-so-free) Chilean water model: the case of the Antofagasta Region, Atacama Desert, Chile, Extr. Ind. Soc. In Press. https://doi.org/10.1016/j.exis.2022.101081

## Value of the Data

• The Chilean water model has been described as an extreme case of resource privatization. This model has led to a high level of conflict between multiple water users in the Atacama Desert. The shared data would allow researchers to appreciate the accumulated allocation of water rights amid this complex context, and thus gives an insight into the problems that emerge from this process.
• The data would provide empirical grounds to investigate the distribution of waters in the Atacama Desert, before and after the implementation of the neoliberal Chilean water model.
• The data would be useful for researchers interested in the Chilean water model, decision makers working on water issues, professionals from the public sector, non-governmental organizations interested in the sustainability of water use, social movements involved in problems relating to water, and for the population in general.
• To reuse the data, it is key to pay attention to variations of the allocated water flows in time, comparing how water rights are assigned to different uses and basins in certain historical moments. To do so, it is useful to display the accumulated water flows as a function of time (years).
• The dataset could be particularly useful when related to historical and political processes associated with water management. Thus, it is possible to understand the relation between particular governance contexts and their effects on the distribution of water.

## 1 Data Description

Our final dataset consists of a single Excel file that we created to characterize the evolution of the accumulated allocation of water rights within the Antofagasta Region between 1905 and 2018, measured in liters per second (L/s). The data are part of a related research article that analyzed the water dispossession and privatization process in the region, particularly in relation to changes in state regulations, water governance, and the growth of mining and other extractive industries [1]. The Excel file in question has two sheets. The first sheet (“Data”) contains 115 rows, one for the top header and the rest for the 114 years following 1905 (until 2018). For each year, the 16 columns that follow the left header describe the accumulation of water rights granted (L/s), for each year, under different variables. These variables are specified in the second sheet (“Metadata”) and also presented in Table 1. There, the codes that are used in the sheet “Data” are put in the first column and then explained in the second column [2].

**Table 1:** Description of the codes used in the dataset to name the different variables for which we calculated the accumulated water flow.

Code of the variable	Description	Column in the Excel file
Year	Year when the water rights are granted	A
LB	Total water flow granted in the Loa Basin (L/s)	B
SAB	Total water flow granted in the Atacama Salt Flat Basin (L/s)	C
LBU	Groundwater flow granted in the Loa Basin (L/s)	D
LBS	Superficial water flow granted in the Loa Basin (L/s)	E
ASFU	Groundwater flow granted in the Atacama Salt Flat Basin (L/s)	F
ASFS	Superficial water flow granted in the Atacama Salt Flat Basin (L/s)	G
LB1	Total water flow granted in the Loa Basin for sanitary use (L/s)	H
LB2	Total water flow granted in the Loa Basin for industrial use (L/s)	I
LB3	Total water flow granted in the Loa Basin for mining (L/s)	J
LB4	Total water flow granted in the Loa Basin for other uses (L/s)	K
LB5	Total water flow granted in the Loa Basin for irrigation (L/s)	L
SA1	Total water flow granted in the Atacama Salt Flat Basin for sanitary use (L/s)	M
SA2	Total water flow granted in the Atacama Salt Flat Basin for industrial use (L/s)	N
SA3	Total water flow granted in the Atacama Salt Flat Basin for mining (L/s)	O
SA4	Total water flow granted in the Atacama Salt Flat basin for other uses (L/s)	P
SA5	Total water flow granted in the Atacama Salt Flat Basin for irrigation (L/s)	Q

## 2 Experimental Design, Materials and Methods

We elaborated the dataset according to the following procedures:

1. The initial data from the official public records on water rights had information about 1047 water rights, described according to 65 variables. We reviewed and validated each one of them in order to avoid errors in the data, inconsistencies between the data, or/and the absence of relevant information. To verify this, we compared the data with bibliographic sources and interviewed public officials. First, to identify incomplete registers, those water rights from the initial database which lacked essential information (i.e., owner of the water right, water flow, catchment location) were recognized. Second, to identify misleading registers in the initial dataset, we compared it with literature that surveys the allocation of water rights in the Antofagasta Region [3], [4], [5]. Accordingly, some water rights from the initial source would not appear in the literature, or would present relevant inconsistencies. Both incomplete and misleading registers added up to 25 cases, which we eliminated. Third, through the interviews with public officials, we validated the suitability of such a method to discard registers . As it is not unusual to have errors in the official public records, which therefore should be contrasted with the relevant literature in order to increase the certainty of the data, the interviewees approved the described procedure. Fourth, for those water rights from the dataset that were not described according to their water use, we inferred this information from the name and productive sector related to the respective owners (irrigation, mining, industry, drinking water supply, etc.). After this process, the dataset came to be composed of 1022 water rights, adding up to a total water flow of 28,349 L/s.
2. Then, we synthesized the data down to those variables (describing each water right) that were relevant to the related research article [1]: (a) annual average of the granted water flow (L/s), (b) year of granting, (c) type of water (groundwater or superficial water), (d) use of the water (sanitary, industrial, for mining, irrigation, other uses) and (e) location (basin where the catchment occurs).
3. After the aforementioned synthesis, in order to characterize the distribution of allocated water rights (L/s) in relation to their location (basin), we calculated the accumulated water flow for each basin. Accordingly, the Loa River and the Atacama Salt Flat watersheds concentrated 67% of the allocated water flow in the Antofagasta Region (41% the former and 26% the latter). Therefore, we decided that it would be appropriate to narrow the scope down to these two basins within the region.
4. Thus, the production of the final data involved the calculation of the accumulated waterflow (L/s) allocated each year under different variables (i.e., ground or surface water, water use), in the basins of the Loa River and Atacama Salt Flat. The year when each water right in the initial database was granted allowed us to sum the accumulated water flows for each year between 1905 and 2018. We analyzed this for (a) the total accumulated water flow allocated in each basin, (b) the accumulated water flow according to the type of water (ground or surface water) for each basin, and (c) the accumulated water flow according to water use (sanitary, industrial, for mining, irrigation, or other uses) for each basin.
5. The aforementioned process allowed us to create the final dataset, which is composed of 17 variables (Table 1) that describe the different conditions under which  we calculated the accumulated water flow (granted as water rights) for each year. Table 2 illustrates the process described above.

**Table 2:** Illustrative explication of the calculation of the accumulated water flows for each year.

Year	LB (Loa Basin)	SAB (Salt Flat Atacama Basin)
1905	Water flow allocated in 1905 (L/s) in the LB	Water flow allocated in 1905 (L/s) in the SAB
1906	Water flow allocated in 1905 in the LB (L/s) + Water flow allocated in 1906 in the LB (L/s)	Water flow allocated in 1905 (L/s) in the SAB + Water flow allocated in 1906 (L/s) in the SAB
1907	Water flow accumulated between 1905 and 1906 in the LB (L/s) + Water flow allocated in 1907 in the LB (L/s)	Water flow accumulated between 1905 and 1906 in the SAB (L/s) + Water flow allocated in 1907 in the SAB (L/s)
1908	Water flow accumulated between 1905 and 1907 in the LB (L/s) + Water flow allocated in 1908 in the LB (L/s)	Water flow accumulated between 1905 and 1907 in the SAB (L/s) + Water flow allocated in 1908 in the SAB (L/s)
Until 2018	The proceeding is repeated until 2018	The proceeding is repeated until 2018

Finally, it is important to add that after doing the research that led to the production of this dataset, we updated it with new information and/or corrections done by the Chilean Water Agency to the official public records on water rights (initial database). Thus, there might be minor differences between the data in the original publication [1] and the data shared through this article [2]. Notwithstanding, this does not change what we published in the related research article [1].

## Ethics Statements

The initial data has public access within the Chilean regulation.

## CRediT authorship contribution statement

**Matías Calderón-Seguel:** Conceptualization, Methodology, Validation, Formal analysis, Investigation, Data curation, Writing – original draft, Writing – review & editing, Visualization. **María Christina Fragkou:** Conceptualization, Validation, Supervision, Project administration, Funding acquisition. **Rodrigo Fuster:** Conceptualization, Validation, Formal analysis. **Francisco Mayol:** Writing – original draft, Writing – review & editing. **Manuel Prieto:** Conceptualization, Methodology, Validation, Investigation, Writing – review & editing, Supervision, Project administration, Funding acquisition.

## Declaration of Competing Interest

The authors declare that they have no known competing financial interests or personal relationships that could have appeared to influence the work reported in this paper.

## Data Availability

Accumulative allocation of water rights in the Atacama Desert (Antofagasta Region, Chile), 1905–2018 (Original data) (Mendeley Data).
